# Targeted distribution of nicotine patches by mail to rural regions of Canada: Predictors of patch use

**DOI:** 10.18332/tpc/197456

**Published:** 2025-01-24

**Authors:** Christina Schell, Alexandra Godinho, Michael Chaiton, Scott T. Leatherdale, John A. Cunningham

**Affiliations:** 1 Institute for Mental Health and Policy Research, Centre for Addiction and Mental Health, Toronto, Canada; 2 Humber River Health, Toronto, Canada; 3 Dalla Lana School of Public Health, University of Toronto, Toronto, Canada; 4 School of Public Health and Health Systems, University of Waterloo, Waterloo, Canada; 5 Department of Psychiatry, University of Toronto, Toronto, Canada; 6 National Addiction Centre, Institute of Psychiatry, Psychology and Neuroscience, King’s College London, London, United Kingdom

**Keywords:** rural, tobacco cessation, nicotine replacement therapy, mass distribution

## Abstract

**INTRODUCTION:**

Rural regions generally report higher smoking rates than urban centers, which increases the risk of tobacco related harms and consequences, and makes promoting smoking cessation in these areas a priority. Mass distribution of nicotine replacement therapy (NRT) by postal mail has been found to increase the odds of successful cessation attempts. Understanding factors that contribute to the use of NRT could help maximize this intervention’s effectiveness.

**METHODS:**

People who smoke cigarettes and live in rural areas of Canada were recruited from December 2020 to February 2022 using random digit telephone dialing. Participants were either randomized to be mailed a free, 5-week supply of NRT patches (experimental condition; n=252) or not (control condition; n=246). This secondary analysis used data from this randomized controlled trial to conduct an ordinal regression to determine if any variables measured at baseline predicted which participants in the experimental condition used none, some, or all of the NRT patches.

**RESULTS:**

Greater confidence in ability to quit (AOR=1.07; 95% CI: 1.00–1.15) independently predicted more patch use, while living in more remote places (AOR=0.25; 95% CI: 0.07–0.90) and past substance use (compared to having no history) (AOR=0.68; 95% CI: 0.45–1.04) independently predicted less use.

**CONCLUSIONS:**

Understanding what contributes to NRT use in rural mass distribution programs could help maximize the odds of successful cessation attempts, personalize treatment recommendations, and target limited rural resources. Future research focused on rural NRT use and smoking cessation is merited.

## INTRODUCTION

Individual health generally decreases with greater distance from urban centers^1^ and with over 17% of Canadians living in areas designated rural, remote and Northern^2^, public health interventions aimed at improving the urban-rural health disparity are needed. Addressing the issue of rural health inequity is complicated by a number of unique barriers (e.g. low population density, large geographical distances, and limited access to health professionals and programs)^3,4^ . One modifiable risk factor is in the promotion of healthy behavior change^5^, including smoking cessation^6^. Rates of tobacco use tend to be higher in rural areas^7^ and as tobacco is a leading cause of preventable disease, morbidity, and mortality^8^, smoking cessation can greatly reduce the risk of experiencing associated harms and consequences, and could help improve health outcomes in rural communities^7^.

The accepted first-line pharmacotherapy for smoking cessation is nicotine replacement therapy (NRT)^9^. NRT comes in different forms (e.g. gum, transdermal patch, nasal spray), has few contraindications, and can increase the chances of successful quit attempts^9^. Due to its relative safety and well-established effectiveness, mass distribution programs provide NRT by postal mail to smokers interested in quitting^10^. In randomized controlled trials (RCTs) of this intervention, participants randomized to receive patches have reported greater odds of successful cessation compared to participants not randomized to receive patches^10-12^. One RCT conducted with a general population sample of Canadians reported that during the 6-month follow-up survey, the odds that participants who were mailed patches would report not smoking in the previous 30-days (30-day point prevalence abstinence) were over two times higher compared to those who were not mailed NRT (OR=2.65; 95% CI: 1.44–4.89)^13^. Furthermore, a secondary analysis of the data found a larger effect among rural versus urban participants (rural OR=9.59; 95% CI: 1.19–77.16; urban OR=2.16; 95% CI: 1.12–4.15)^14^. This finding was supported by a RCT which used the same procedure, but specifically targeted rural Canadians. In this rural sample, 30-day point prevalence abstinence at 6 months was over three times greater among participants who were mailed patches (OR=3.29; 95% CI: 1.52–7.13)^15^.

As NRT use is correlated with greater cessation success^9^, using the amount prescribed for the recommended duration is an important aspect of mass distribution initiatives; however, considerable variation has been reported in the number of participants that report using at least some of the NRT provided (40–90%)^16^. Understanding factors that contribute to use may provide an opportunity to improve this already promising intervention. A number of possible predictors have been identified in general and clinical samples including, demographics (e.g. education level, age, employment)^16-18^, individual factors (e.g. motivation, forgetfulness, attitudes towards quitting and towards NRT)^17,18^, clinical characteristics (e.g. level of nicotine dependence, withdrawal symptoms, previous NRT use)^16-18^ and concurrent concerns, including mental health and other addictions^17,19^. Beyond the individual level, variables including social support^17^, life events, and environmental triggers (e.g. friends or family that smoke)^18,19^ have also been implicated.

While a number of contributing factors have been identified, limited literature has included place of residence. Rural-urban comparisons consistently note a number of differences which could impact the generalizability of these predictors^5^. Understanding which variables predict use in rural settings could help personalize treatment recommendations, increase the likelihood of successful cessation attempts, and target limited resources. The current secondary analysis investigated which variables, if any, predicted which participants in a rural sample used all, some, or none of the NRT patches mailed to them.

## METHODS

### Randomized controlled trial (RCT)

Details of the original RCT and outcomes have been published elsewhere^7,15^. Briefly, people who smoke cigarettes were recruited from across Canada via random digit telephone dialing which targeted rural regions. Rural areas were defined as areas with a population <1000 and outside the commuting zone (travel time <150 minutes)^20^ of larger urban centers (populations of ≥10000)^7^. Individuals living in these areas were eligible to participate if they were aged ≥18 years, smoked ≥10 cigarettes per day, were willing to complete two surveys 6 months apart, and indicated interest in receiving and using free NRT patches, if offered. Exclusion criteria included health contraindications for using NRT without doctor supervision (i.e. heart or circulation problems, pregnancy, currently breastfeeding, and/or allergy to tape). Recruitment occurred December 2020 to February 2022^7,15^.

Using a 1:1 ratio, participants were randomized to the no intervention control or the experimental condition. Those in the experimental group received a free, 5-week supply of NRT patches by mail. All participants completed the baseline survey which collected information about their smoking and demographic characteristics. A second survey was conducted 6 months after enrollment^7,15^.

### Measures

*NRT patch use*

During the 6-month follow-up survey, participants in the intervention condition were asked how much of the NRT patches they used. Response options for this self-reported outcome were limited to none, some, or all. In the analysis, the responses were dummy coded 0, 1, and 2, respectively, and treated as an ordinal variable.

*Remoteness index*

While the Canadian population is unevenly distributed across the country, health services tend to be concentrated in larger urban centers^21^. This can result in two similarly sized rural communities experiencing unequal access to healthcare and resources due to their proximity to larger communities. To help account for ease of access, Statistics Canada developed the Remoteness Index (RI) by weighing a community’s size, population density, and proximity to larger population centers. The resulting values range 0 to 1 and can be used as a proxy to estimate service access, with smaller values indicating easier access and higher values indicating greater remoteness^20^. Different methods may be used to subdivide the scale into more meaningful categories and while the continuous index will be used in all models, categories derived from the manual method will be included for descriptive purposes [i.e. easily accessible areas (<0.1500), accessible areas (0.1501–0.2888), less accessible areas (0.2889–0.3898), remote areas (0.3899–0.5532), and very remote areas (>0.5532)]^22^. RI scores for each participant were determined using their mailing address^23^.

*Smoking characteristics*

In addition to demographic variables, measures of self-reported smoking behavior were collected (i.e. number of cigarettes smoked per day, years smoking daily, number of previous attempts to quit smoking). Degree of nicotine dependence was determined through administration of the 6-item Fagerström test for nicotine dependence (FTND). Total scores range 0 to 10 and can be categorized as low (0–2), low-medium (3–4), medium (5), high (6–7) and very high (8–10) levels of dependence^24^. Self-reported confidence in ability to quit and the importance of quitting were assessed on a 10-point Likert scale ranging from low confidence/not important (1) to very confident/very important (10) Finally, the Transtheoretical Model was used to categorize participants according to their readiness to quit smoking. Three stages were identified in the sample: pre-contemplation, contemplation, and preparation^25^.

*Other predictors*

Participants were asked about any previous or current psychiatric history (e.g. depression, anxiety, schizophrenia, bipolar disorder, personality disorder, and/or attention deficit hyperactivity disorder) and past or current substance use history (e.g. marijuana, cocaine, sedatives, opiates, stimulants and/or other drugs). Quality of life was assessed using the 8-item EUROHIS QOL scale, which asks respondents to rate their satisfaction with different aspects of life (e.g. health, relationships, financial means, place of residence). Total scores range 0–40 with higher values indicating greater perceived quality of life^26^. Finally, self-reported frequency of alcohol use, frequency of having ≥5 drinks on one occasion (binge drinking), and number of drinks consumed on a typical day when drinking, were collected.

### Secondary analysis

A literature review identified many predictors reported in other research as possibly being related to over-the-counter NRT use in general and clinical samples^16^. Due to sample size limitations, it was not feasible to include all the variables identified by the review in a multivariate regression, and purposeful selection was used to reduce the number of predictors for analysis. To accomplish this, a series of univariate ordinal regressions were conducted between each variable of interest and the dependent variable (amount of NRT used). Analyses involving an independent variable with p>0.25 were excluded, the variable being unlikely to have a relationship with NRT use. The remaining potential predictors were included in the multivariate ordinal regression^27^.

All three levels of patch use (all, some, none) were retained in order to reduce the loss of information^28^ and complementary log-log link function was used to account for the uneven distribution of participants between levels of the dependent variable^29^. Participants who reported not using any patches served as the reference group. All analyses were conducted using SPSS version 27.0^30^.

### Ethics approval

Verbal consent to participate was obtained by telephone and categories were compensated for the completion of each interview. The design of the study and all research methods were approved by the ethics review committee at the Centre for Addiction and Mental Health.

## RESULTS

### Sample characteristics

Of the 498 participants, 252 were randomized to the experimental condition. The follow-up rates at 6 months were good, with 84.1% of the experimental group completing the survey. This analysis was limited to the 201 participants who reported receiving the patches. The total sample had a mean age of 58.1 years (SD=12.8), was 54.0% male, 54.2% were married or common-law, 53.5% were employed full- or part-time, and 31.3% had a household annual income ≥CND 60000 (1 Canadian dollar about US$0.69). On average, participants had been smoking ≥10 cigarettes per day for 32.0 years (SD=17.3), smoked 19.0 (SD=10.6) cigarettes per day, and only 5.5% reported never previously trying to quit smoking. Slightly less than half of the sample (41.3%) reported never using other substances and over half (65.7%) reported no history of psychiatric diagnosis. In total, the sample reported drinking 3.1 drinks (SD=2.8) on a typical day when they were drinking, 22.0% reported never drinking ≥5 drinks on one occasion, and 20.9% reported never drinking alcohol. The average quality of life score was 29.7 (SD=6.0; max=60) and the average Remoteness Index for the sample was 0.4040 (SD=0.1404), which can be categorized as ‘remote’^22^ [two participants (0.01%) did not supply a rural address, but were retained in the analysis]. Additional variables are presented in Table 1, as well as frequency and descriptive statistics stratified by the amount of NRT patches used (as reported at follow-up at 6 months).

**Table 1:** Descriptive analyses of a sample of Canadians who smoke and live in rural regions, stratified by the dependent variable: amount of mailed nicotine replacement therapy patches used (N=201)

*Characteristics*	*Total (N=201)^a^ % (n)*	*Amount of NRT patch use*		
		*All (N=42) % (n)*	*Some (N=109) % (n)*	*None (N=50) % (n)*
**Remoteness index**, mean (SD)	0.4040 (0.1404)	0.3603 (0.1218)	0.4213 (0.1345)	0.4031 (0.1609)
**Demographic**				
Age (years), mean (SD)	58.1 (12.8)	59.2 (13.1)	57.2 (13.1)	59.1 (11.7)
Male	54.0 (108)	63.4 (26)	48.6 (53)	58.0 (29)
Married/common-law	54.2 (109)	50.0 (21)	57.8 (63)	50.0 (25)
Employed full/part-time	53.5 (107)	45.3 (19)	53.7 (58)	60.0 (30)
Household annual income (≥CND 60000)	31.3 (60)	29.3 (12)	31.4 (32)	32.7 (16)
**Education level**				
<High school	30.8 (62)	31.0 (13)	30.3 (33)	32.0 (16)
High school	38.3 (77)	40.5 (17)	36.7 (40)	40.0 (20)
Any post-secondary	30.8 (62)	28.6 (12)	33.0 (36)	28.0 (14)
**Smoking status**				
Cigarettes/day, mean (SD)	19.0 (10.6)	20.6 (9.3)	18.6 (12.6)	18.8 (6.1)
Daily smoking (years), mean (SD)	32.0 (17.3)	36.5 (17.8)	30.3 (16.9)	32.2 (17.5)
**Nicotine dependence** ^b^				
Low	13.3 (26)	19.0 (8)	6.8 (7)	55.6 (25)
Low-moderate	33.3 (65)	26.2 (11)	39.2 (40)	31.1 (14)
Moderate	47.7 (93)	42.9 (18)	49.0 (50)	11.1 (5)
High	5.6 (11)	11.9 (5)	4.9 (5)	2.2 (1)
**Previous quit attempts**				
0	5.5 (11)	2.4 (1)	6.4 (7)	6.0 (3)
1–2	21.9 (44)	11.9 (5)	23.9 (26)	26.0 (13)
3–5	36.8 (74)	42.9 (18)	33.9 (37)	38.0 (19)
≥6	35.8 (72)	42.9 (18)	35.8 (39)	30.0 (15)
**Confidence in ability to quit**, mean (SD)	5.7 (2.7)	5.9 (2.9)	5.9 (2.7)	5.0 (2.5)
**Importance of quitting**, mean (SD)	7.2 (2.5)	7.2 (2.7)	7.5 (2.4)	6.5 (2.6)
**Stage of change**				
Pre-contemplation	28.4 (57)	23.8 (10)	27.5 (30)	34.0 (17)
Contemplation	34.3 (69)	33.3 (14)	33.0 (36)	38.0 (19)
Preparation	37.9 (75)	42.9 (18)	39.4 (43)	28.0 (14)
**Psychiatric history**				
None	65.7 (132)	76.2 (32)	63.3 (69)	62.0 (31)
Past only	14.9 (30)	9.5 (4)	16.5 (18)	16.0 (8)
Current	19.4 (39)	14.3 (6)	20.2 (22)	22.0 (11)
**Substance use history**				
None	41.3 (83)	57.1 (24)	40.4 (44)	30.0 (15)
Past only	25.4 (51)	11.9 (5)	28.4 (31)	30.0 (15)
Current	33.3 (67)	31.0 (13)	31.2 (34)	40.0 (20)
**Frequency of alcohol use**				
Never	20.9 (42)	26.2 (11)	19.3 (21)	20.0 (10)
Monthly or less	23.9 (48)	19.0 (8)	29.4 (32)	16.0 (8)
2–4 times/month	18.9 (38)	16.7 (7)	21.1 (23)	16.0 (8)
2–3 times/week	15.9 (32)	19.0 (8)	12.8 (14)	20.0 (10)
≥4 times/week	20.4 (41)	19.0 (8)	17.4 (19)	28.0 (14)
**Frequency of ≥5 drinks on one occasion**				
Never	22.0 (44)	23.8 (10)	24.1 (26)	16.0 (8)
Monthly or less	45.5 (91)	50.0 (21)	46.3 (50)	40.0 (20)
2–4 times/month	18.5 (37)	16.7 (7)	17.6 (19)	22.0 (11)
2–3 times/week	4.0 (8)	2.4 (1)	5.6 (6)	2.0 (1)
≥4 times/week	10.0 (20)	7.1 (3)	6.5 (7)	20.0 (10)
**Number of drinks on a typical day**, mean (SD)	3.1 (2.8)	2.5 (2.3)	3.1 (3.0)	3.6 (2.8)
**Quality of life**, mean (SD)	29.7 (6.0)	29.2 (6.0)	30.2 (5.7)	29.1 (5.5)

NRT: nicotine replacement therapy.

a Sample sizes vary due to missing data on some variables.

b Fagerström test for nicotine dependence (FTND). CND: Canadian dollars.

### Predictors of nicotine patch use

A total of 8 variables (i.e. RI, employment status, number of years smoking daily, FTND categories, number of previous quit attempts, confidence in ability to quit, substance use history, and number of alcoholic drinks consumed on a typical day) were found to have a potential relationship with the amount of patches used (p≤0.25). Results for all the ordinal regressions are presented in Table 2.

**Table 2:** Univariate ordinal logistic regressions predicting the amount of mailed nicotine replacement therapy patches used (all, some, or none) in a sample of Canadians who smoke and live in rural regions (N=201)^a^

*Variable*	*χ^2^ (df)*	*p*	
Remoteness index	4.03 (1)	0.045	*
Age (years)	0.09 (1)	0.765	
Gender	0.87 (1)	0.352	
Marital status	0.09 (1)	0.769	
Employment status	1.90 (1)	0.168	*
Household annual income	0.12 (1)	0.730	
Education level	0.04 (2)	0.983	
Cigarettes/day	0.92 (1)	0.337	
Daily smoking (years)	2.41 (1)	0.121	*
Nicotine dependence^b^	5.39 (3)	0.145	*
Previous quit attempts	4.80 (3)	0.187	*
Confidence in ability to quit	1.32 (1)	0.250	*
Importance of quitting	0.75 (1)	0.387	
Stage of change	1.69 (2)	0.429	
Psychiatric history	2.60 (2)	0.273	
Substance use history	8.17 (2)	0.017	*
Frequency of alcohol use	1.10 (4)	0.894	
Frequency of ≥5 drinks on one occasion	3.45 (4)	0.486	
Number of drinks on a typical day	3.30 (1)	0.069	*
Quality of life	0.07 (1)	0.792	

Complementary Log-Log link function.

* Included in multivariate model.

a Sample sizes vary due to missing data on some variables.

b Fagerström test for nicotine dependence (FTND).

The 8 variables identified by the univariate analyses were entered into the multivariate ordinal logistic regression as predictors to determine their effect on NRT patch use. Missing data resulted in the listwise deletion of 10 participants resulting in a final sample of 191 for the multivariate model. The analysis found living in more remote places decreased the odds of using more NRT (AOR=0.25; 95% CI: 0.07–0.90). In contrast, an increased confidence in the ability to quit smoking was associated with an increase in the odds of using more NRT patches (AOR=1.07; 95% CI: 1.00–1.15). Substance use history also had a significant effect on prediction of NRT use (p=0.015). Post hoc comparisons revealed that having a past history of substance use decreased the odds of using more NRT (AOR=0.68; 95% CI: 0.45–1.04) compared to those with no history of substance use; however, no significant effect was found in the other pair-wise comparisons (current vs no history, p=0.079; current vs past history, p=0.209). Regression estimates for all variables are presented in Table 3.

**Table 3:** Multivariate ordinal logistic regressions with factors predicting the amount of mailed nicotine replacement therapy patches used (all, some or none) in a sample of Canadians who smoke and live in rural regions (N=191)

*Variable*	*B (SE)*	*p*	*AOR (95% CI)*
Employment^a^	-0.11 (0.20)	0.581	
Remoteness index	-1.39 (0.66)	0.035	0.25 (0.07–0.90)
Daily smoking (years)	0.003 (0.006)	0.560	
Confidence in ability to quit	0.07 (0.03)	0.040	1.07 (1.00–1.15)
**Nicotine dependence**^b^ (Ref. High)		0.154	
Low	-0.52 (0.49)	0.297	
Low-moderate	-0.79 (0.45)	0.077	
Moderate	-0.90 (0.44)	0.042	
**Previous quit attempts** (Ref. ≥6)		0.169	
0	-0.43 (0.41)	0.297	
1–2	-0.51 (0.24)	0.036	
3–5	-0.12 (0.21)	0.580	
**Substance use history** ^c^		0.015	
Current (Ref. none)	-0.38 (0.22)	0.079	-
Past only (Ref. none)	-0.67 (0.23)	0.004	0.68 (0.45–1.04)
Current (Ref. past only)	0.29 (0.23)	0.209	-
**Number of drinks on a typical day**	-0.03 (0.03)	0.349	

AOR: adjusted odds ratio. SE: standard error. Ref: reference.

a Factors: full/part-time, unemployed/homemaker/student.

b Fagerström test for nicotine dependence (FTND).

c Self-reported, past or current use (includes marijuana, cocaine, sedatives, opiates, stimulants and other drugs).

## DISCUSSION

Mass distribution of NRT without behavioral support requires fewer resources than many other interventions, but still increases the odds of successful quit attempts among the general population and rural samples^13,15^. Such programs create an opportunity to provide many at-risk individuals with access to effective treatment, thereby improving individual health and reducing disease burden. Exploring ways to increase this intervention’s effect while conserving limited resources is especially merited in rural settings^21^.

While there is a large body of work identifying potential predictors of NRT use, less is known about the generalizability to rural populations. This research identified confidence in ability to quit smoking, substance use history, and remoteness as predictors of patch use in this rural sample. Additional research is needed to support this preliminary work and investigate if these, or other factors, could be used to increase the effectiveness of mass NRT distribution by targeting slightly modified versions in order to meet the needs of more individuals.

Identifying remoteness as a predictor of NRT use supports recommendations that research consider the impact that area of residence has on health^5^. It is also not uncommon for positive treatment effects found in general population or urban samples to decrease or disappear when programs are trialed in rural communities^7,31^. Thus, the current finding that NRT use decreased with greater remoteness could suggest that providing a modified version of the mass distribution intervention (e.g. with behavioral support) may be more beneficial to those living in more remote communities. However, program evaluations will need to weigh any increased effect against potentially greater costs given the limited resources typically found in very remote areas^5,6^. In this respect, digital interventions (e.g. delivered via internet, cellular/telephones, apps) may be solutions deserving further research^31,32^. Alternately, although there is evidence supporting the effectiveness of mass distribution initiatives, there is also some research reporting no significant treatment effect of NRT use in rural samples^8^, which could indicate the need for entirely different treatment options.

### Strengths and limitations

Research concerning NRT use in rural samples is limited and results should be interpreted with some caution. In particular for the current analysis, sample size limitations, the self-reported nature of the data, and the secondary nature of the analyses need to be considered. First, larger samples are recommended for future investigations of NRT use in order to accommodate the inclusion of a larger number of potential predictors, interaction terms and increase the ability to detect small effects. Nevertheless, the procedure used to target rural areas was successful and resulted in a subsample of rural Canadians (n=201) with nearly half of the participants living in communities that can be considered remote (29.9%) and very remote (16.9%). Compared to the general population, only 3.7% and 0.7% of Canadians live in these areas, i.e. remote and very remote, respectively^2^. Therefore, despite some limitations, this sample provides a unique opportunity to better understand an often-underrepresented group.

The results must also be interpreted with caution due to the self-reported nature of the data, as the use of the NRT patches may have been over- or under-reported. Most participants (54%) reported using ‘some’ of the NRT patches. While expected, this category also has the greatest risk of biased reporting as it is possible, for example, that some participants who used all of the NRT patches may misremember and only report ‘some’ use, while other participants who did not use any patches may report ‘some’ use in order to respond more favorably. While self-report bias is a concern, particularly in the ‘some’ response category, retaining the information and not dichotomizing the variable is a definite advantage of this analysis. Indeed, the assumption of proportional odds in the ordinal logistic regression ensures that the effect of the predictors is consistent across different thresholds^28^. That is, the odds of using ‘none’ versus using ‘some’ or ‘all’ of the patches is the same as the odds of using ‘none’ and ‘some’ of the patches versus using ‘all’. Thus, while future projects may consider adding methods to validate the self-reported patch use, not dichotomizing variables in order to retain as much information and variation as possible, is strongly recommended.

### Implications for future research

Except for the remoteness index, this analysis primarily focused on individual level characteristics. It is recommended that future research include a mix of interpersonal (e.g. number of friends and family that smoke)^18,19^, community (e.g. social capital, population density)^5^ and system level (e.g. few health professionals, limited public transportation)^3,4^ social determinants of health, and more direct measures of resource availability and use. Rural communities are diverse and not all communities are impacted in the same way^5^. Given the noted impact of social capital on health and its importance in rural culture^5,33^, addressing community level variation could increase the effectiveness of interventions. Indeed, socioeconomic characteristics and demographics are estimated to contribute to nearly two-thirds of community level variation in smoking rates^34^. Understanding these differences could allow health promotion efforts to customize interventions at a community level, as well as create more personalized treatments for individuals.

Future research should ask participants why they used, or did not use, the NRT patches in order to assess if use/non-use was intentional. Although the correlation between NRT use and successful cessation is well documented^9^, the causal relationship remains unclear. That is, it is uncertain whether a relapse to smoking causes individuals to stop using NRT or if discontinuing NRT causes a relapse to smoking^35^. Including appropriate questions in future studies will help better understand the reasons and conditions behind unsuccessful quit attempts. Health promotion efforts could then work to reduce factors that contribute to relapse and/or promote factors that improve use and create more personalized approaches to improve treatment outcomes for individuals interested in quitting smoking.

## CONCLUSIONS

Addressing the higher rates of tobacco use in rural areas is an important public health concern and promoting smoking cessation can help reduce tobacco related disease burden which could subsequently reduce urban-rural health disparities. Improving the use of NRT as prescribed (i.e. amount and duration) is one modifiable aspect of this promising intervention which could further increase the odds of successful quit attempts. The three predictors of NRT use identified in this preliminary work suggest ways to modify the mass distribution intervention to produce a greater treatment effect while conserving limited resources and provide more rural smokers with access to an effective treatment option^7,8^. Additional research focused on people who smoke and live in rural, remote, and Northern communities, is highly recommended.

## Data Availability

The data supporting this research are available from the authors on reasonable request.
